# Revealing −1 Programmed Ribosomal Frameshifting Mechanisms by Single-Molecule Techniques and Computational Methods

**DOI:** 10.1155/2012/569870

**Published:** 2012-04-01

**Authors:** Kai-Chun Chang

**Affiliations:** Institute of Molecular and Cellular Biology, National Taiwan University, Taipei 10617, Taiwan

## Abstract

Programmed ribosomal frameshifting (PRF) serves as an intrinsic translational regulation mechanism employed by some viruses to control the ratio between structural and enzymatic proteins. Most viral mRNAs which use PRF adapt an H-type pseudoknot to stimulate −1 PRF. The relationship between the thermodynamic stability and the frameshifting efficiency of pseudoknots has not been fully understood. Recently, single-molecule force spectroscopy has revealed that the frequency of −1 PRF correlates with the unwinding forces required for disrupting pseudoknots, and that some of the unwinding work dissipates irreversibly due to the torsional restraint of pseudoknots. Complementary to single-molecule techniques, computational modeling provides insights into global motions of the ribosome, whose structural transitions during frameshifting have not yet been elucidated in atomic detail. Taken together, recent advances in biophysical tools may help to develop antiviral therapies that target the ubiquitous −1 PRF mechanism among viruses.

## 1. Introduction to Programmed −1 Ribosomal Frameshifting in Viruses

**Figure 1:**
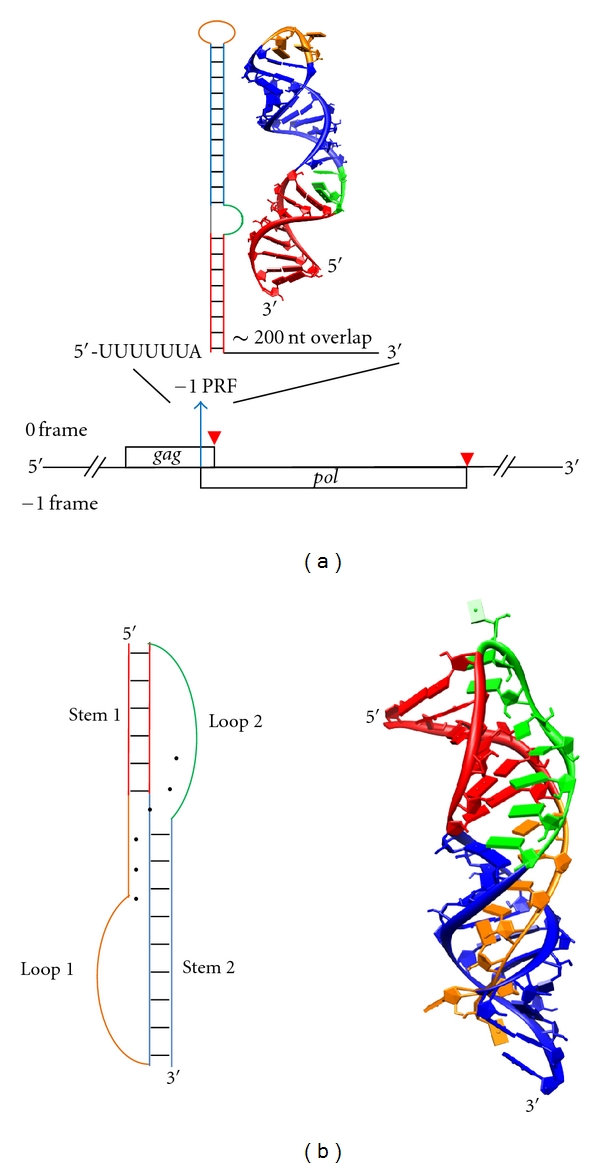
(a) Structure of HIV *gag*/*gag-pol* ORFs, with stop codons indicated by red arrow heads. The* pol* ORF does not contain a start codon and can only be initiated by −1 PRF within the *gag* ORF. Therefore, the two ORFs are said to be “overlapping” by a length of ~200 nt [3]. The secondary and NMR-resolved three-dimensional structures for the −1 PRF-stimulating stem-loop are illustrated with corresponding colors, whereas base pairs are indicated by short black bars [20, 40] (PDB 1Z2J). Stems are shown in red and blue, and loops are shown in orange and green. (b) A minimal (ΔU177) human telomerase pseudoknot adapts a canonical H-type pseudoknot configuration [18, 32] (PDB 1YMO). Tertiary major-groove and minor-groove interactions (base triples) are represented by black dots between bases in the secondary structure depiction. The black dot at the junction of Stem 1 and Stem 2 indicates tertiary interaction between Loop 1 (orange) and Loop 2 (green). This RNA structure is not involved in translational regulation, but rather in the activity of the telomerase complex [18, 41]. Its well-known structure makes it an ideal RNA pseudoknot system for studying frameshifting [32, 42]. Stem 1, Loop 1, Stem 2, and Loop 2 are shown in red, orange, blue, and green, respectively. All 3D molecular representations in this paper were produced using the UCSF Chimera package from the Resource for Biocomputing, Visualization, and Informatics at the University of California, San Francisco, USA (supported by NIH P41 RR001081) [43].

 The genetic information in mRNA is decoded by the ribosome in units of three nucleotides, the codons, being translated into their corresponding amino acids. Consequently, there are three possible reading frames for a given length of nucleotide message. The actual open reading frame starts with the nucleotide triplet AUG and extends with every following triplet being read correctly by the ribosome, ensuring an error rate of frameshifting <3 × 10^−5^ per codon [1, 2]. However, programmed ribosomal frameshifting (PRF) is indispensable for many viruses to regulate their protein expression levels from overlapping ORFs. In human immunodeficiency virus type 1 (HIV-1), −1 PRF occurs at a frequency of 5 to 10% at the junction of *gag* and *pol* genes, resulting in a 20 : 1 to 10 : 1 molar ratio of the structural (Gag) to enzymatic proteins (Gag-Pol polyprotein) (Figure 1(a)) [3–6].

 The viral −1 PRF site contains three characteristic RNA elements [7–12]. (i) A slippery site with the form of X-XXY-YYZ (the dashes separate in-frame triplets), where XXX can be any homopolymeric sequence, YYY can be either AAA or UUU, and Z can be A, U or C. The slippery sequence allows effective base-pairings between the ribosome-bound tRNAs and mRNA even after frameshifting to XXX-YYY (−1 frame). (ii) A 5- to 10-nucleotide-long spacer between the slippery site and the downstream RNA structure. (iii) A downstream pseudoknot or stem-loop (also referred to as a hairpin) structure that is generally thought to act as a “roadblock” to stall the ribosome and subsequently promote ribosome backward slippage.

 Most retroviruses adapt pseudoknots as their −1 PRF-stimulating elements [13–15]. A typical hairpin- (H-) type pseudoknot is characterized by base-pairing between a hairpin loop and a single-stranded region outside that loop (Figure 1(b)) [13, 16]; that is, as shown in Figure 1(b), Stem 2 is formed by base-pairing Loop 1 and the distal end of Loop 2. This brings remote regions of the RNA contour together and gives rise to more complex tertiary interactions, such as base-stacking and triplex base-pairing [17, 18]. As a result, pseudoknots are generally more stable and efficient in promoting −1 PRF, as compared to their stem-loop counterparts with the same compositions of base pairs [14, 17, 19]. Notably, there are still cases like HIV-1 that can utilize simple stem-loops to promote −1 PRF effectively [19, 20].

Subtle alterations in −1 PRF elements have been reported to affect viral production dramatically [21–24]. For HIV-1, despite the intrinsically high mutation rate for RNA viruses, Biswas et al. found that of the 1,000 HIV-1 slippery sequences they obtained, all the UUUUUUA slippery heptamers are exactly identical. Substituting this site with another equivalently efficient slippery sequence, namely, UUUAAAA, can reduce viral titer more than 1,000-fold [4, 25]. More recently, annexin A2 (ANXA2), an eukaryotic multifunctional protein, has been shown to bind the pseudoknot of avian coronavirus infectious bronchitis virus (IBV) and reduce the viral −1 PRF efficiency [26]. Accordingly, the authors suspected that ANXA2 might interact more generally with other viral RNA pseudoknots, thereby acting as an antiviral regulator in eukaryotic cells [26].

 Despite −1 PRF's ubiquity among infectious viruses, and its promising role for serving as an antiviral target [3, 4, 6, 27], the precise molecular mechanism remains elusive. This paper aims to address how recent developments in biophysical tools, specifically single-molecule techniques and computational modeling, can help to elucidate the mechanochemical basis for −1 PRF.

## 2. Single-Molecule Techniques Reveal Mechanochemical Details for Pseudoknot-Stimulated −1 PRF

 Since the diameter of the mRNA entry tunnel in the ribosome is too small to accommodate the dimensions of double-stranded RNA, any RNA secondary structures must be disrupted before being read by the ribosome [28, 29]. One may hence expect that the thermodynamic stability of a downstream mRNA structure should correlate with −1 PRF efficiency, as has been observed in the mRNA strands that contain stem-loop stimulatory structures [19, 30]. Intriguingly, the free-energy change Δ*G* of folded and unfolded pseudoknots measured from UV optical melting profiles does not correlate well with the propensity of frameshifting [13, 18, 31–34]. This discrepancy may be attributed to the fact that thermal melting occurs globally at any base pair, but the ribosome can only unwind the duplex sequentially from 5′ to 3′ end of the mRNA [14]. Due to the unique topology of pseudoknots (Figure 1(b)), the downstream Stem 2 causes supercoiling in the Stem 1 via base-pairing as the ribosome attempts to unwind the 5′-end of Stem 1. Accordingly, Stem 2 must be simultaneously disrupted before allowing ribosome translocation through the entire Stem 1, providing further torsional restraint to the ribosome [31, 35, 36]. In contrast, a simple stem-loop can rotate freely during unwinding. Therefore, other than tertiary interactions, the restriction in rotational freedom explains the superior mechanical stability and −1 PRF efficiency of pseudoknots, compared with energetically equivalent stem-loops [31, 35]. The total unwinding work exerted by the ribosome would thus be larger than the Δ*G* required for just melting the pseudoknots, with some fraction of work dissipated irreversibly [31]. Then, it can be inferred that −1 PRF efficiency should correlate more with the mechanical force required for “pulling” RNA pseudoknots apart, which resembles successive RNA unwinding by the ribosome [7, 14, 31, 32]. Such mechanical pulling of an RNA pseudoknot can be readily carried out by optical tweezers.

 An optical trap is formed by focusing a laser beam to the vicinity of a transparent particle that diffracts the incident light [37, 38]. The particle thus experiences a force from the diffracted photons due to momentum transfer. The intensity profile of the laser beam is chosen to adapt a Gaussian gradient, such that small displacements of the particle (~150 nm) from the beam center produce a counteracting force toward its equilibrium position, acting like a simple harmonic spring. The spring constant, which is determined through control experiments in advance, depends on both the laser profiles and the dielectric properties of trapped objects [37–39].

**Figure 2:**
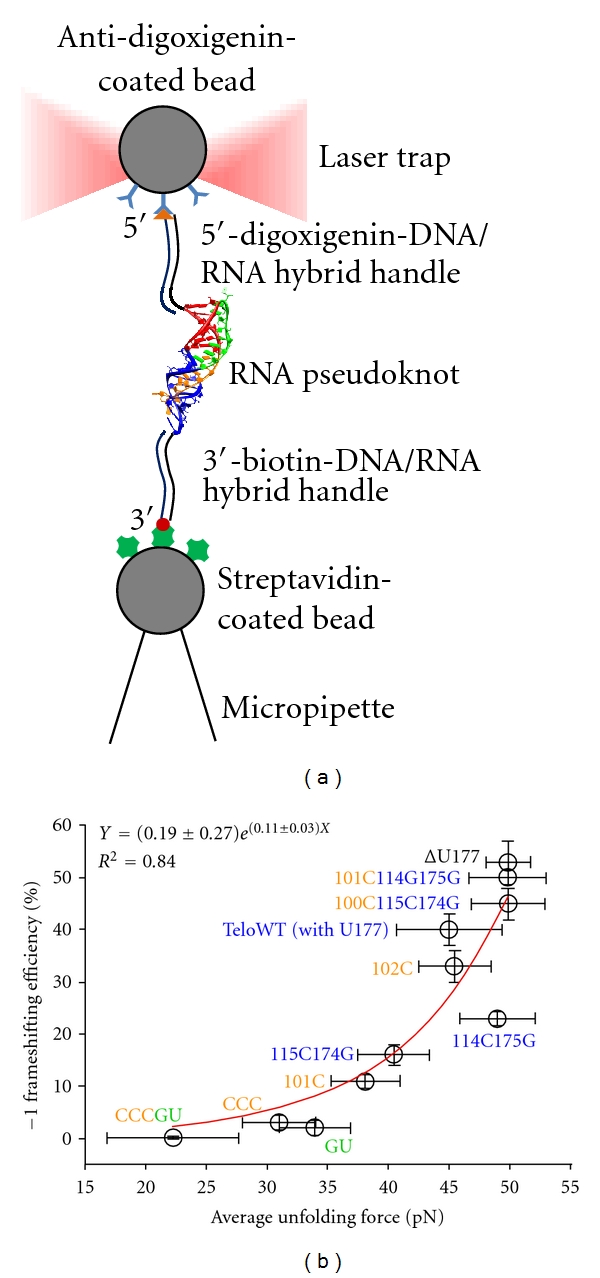
(a) Schematic representation of optical tweezing. The RNA pseudoknot is flanked by DNA handles that are end-labeled with biotin and digoxigenin, respectively. The handles can then attach to surface-modified beads via biotin-streptavidin and digoxigenin-antibody interactions. Finally, a trapping laser and a micropipette that moves away from the trap produce tension force on the construct. (b) Correlation between −1 PRF efficiencies and externally applied unwinding forces for different pseudoknot constructs. For details of each construct, the reader is referred to reference [32]. Circles indicate average values. Error bars for −1 frameshifting efficiencies along the *y*-axis are standard errors of the means from *in vitro* bulk frameshifting assay. Error bars for unfolding forces are standard deviations of the optical tweezing measurements. Frameshifting efficiencies correlate better with unfolding forces (*R* ^2^ = 0.84) than melting free energies of pseudoknots (*R* ^2^ = 0.59, see [32]). (b) is reproduced with permission from [32].

By monitoring and/or manipulating biomolecules individually, single-molecule techniques are able to unveil stochastic behaviors and rare events that are otherwise hidden in the ensemble averages from a “bulk” biochemical assay (“in bulk” for short). To facilitate single-molecule manipulation on optical tweezers, two DNA handles are attached to micron-sized polystyrene beads through biotin-streptavidin and digoxigenin-antibody interactions, respectively [48]. The RNA molecule of interest, usually a stem-loop or pseudoknot, is flanked by the DNA handles. One of the beads is trapped by a laser beam, while the other is pulled by a micropipette or another trapping laser (Figure 2(a)).

 Chen et al. discovered that the unwinding force measured by optical tweezers correlates strongly with the −1 PRF frequency (Figure 2(b)) [32]. Extrapolation of the data predicts that 100%  −1 PRF efficiency would be reached by a pseudoknot with an unfolding force around 57 pN. The authors reasoned that pseudoknots with unfolding force above ~60 pN would completely stall the ribosome and result in an abortive translation [32]. Although this prediction has not been confirmed directly, such “roadblocking” effect has been observed in bulk recently [7].

Consistent with the torsional restraint model [35, 36], a similar experiment conducted by Hansen et al. showed that the work performed by optical tweezers during mechanical unfolding of a IBV-based pseudoknot (501 ± 36 kJ/mol, see PK401 in [31]) is much larger than the theoretically estimated free-energy cost (292 kJ/mol) required for both RNA unfolding and stretching [31], with a significant amount of the performed work dissipated irreversibly. The need for extra energy input makes the pseudoknot more resistant to unfolding by optical tweezers and presumably by ribosomes, when compared with its hairpin counterparts with equivalent base-pairing energies. The model also explains the observation that the length and predicted stability of Stem 1 do not always correlate with the frequency of frameshifting, since the effect of torsional restraint must also be taken into consideration; for example, Napthine et al. found that an IBV-derived pseudoknot with 12 bp Stem 1 (Δ*G*_Stem  1,  theory_ = −67.4 kJ/mol, 55%  −1 PRF) actually stimulates −1 PRF more efficiently than that with a 13 bp Stem 1 (Δ*G*_Stem 1,theory_ = −80.0 kJ/mol, 47%  −1 PRF) [49]. 

 Despite the strong mechanical strength of a pseudoknot, HIV-1 is rather unique in that it utilizes a simple stem-loop as its −1 PRF-stimulating element [20], and, as described above, the −1 PRF efficiency is susceptible to mutations in the UUUUUUA frameshifting site (virtually from ~5% to 0% for some point mutations) [4]. Therefore, the role of an efficient slippery sequence should also be taken into consideration. Indeed, the slippery nature of a poly(U) template has been demonstrated by optical tweezers previously [50].

 While optical tweezers reveal the mechanical properties of mRNAs, single-molecule Förster resonance energy transfer (smFRET) serves as a powerful tool for probing conformational changes of single ribosomes [51–57]. When a laser beam is totally and internally reflected in a microscope, it produces an evanescent wave on the other side of the interface. This evanescent wave decays exponentially with distance, and therefore can only penetrate ~100 nm into the reaction chamber [58, 59]. SmFRET exploits this property to excite only the fluorescent samples immobilized on the surface, greatly reducing the background signal from free fluorescent molecules outside the evanescent field. The immobilized fluorescent molecules appear as diffraction-limited spots that can be visualized and recorded by an electron multiplying charge coupled device (EMCCD) camera. The fluorescence intensities and the corresponding FRET efficiencies of single molecules can thus be measured and calculated individually.

A recent smFRET experiment has utilized fluorescently labeled ribosomes to correlate recurring fluctuations in FRET efficiency to intersubunit conformational changes, which in turn indicate ribosomal translocation events [52]. This design allowed the researchers to observe ribosome slipping at codon resolution, which revealed a small fraction (<2%) of the ribosome traces exhibiting FRET cycles larger than the mRNA coding length of the homopolymeric poly(U) template. In contrast, additional FRET cycles that are indicative of ribosomal slippage, were not observed when a heteropolymeric template was translated [52]. These results again confirm the notion that the U-rich sequence is very slippery [25].

Although a recent study suggests that stem-loops can serve as efficient −1 PRF stimulators, the experiment has utilized a slippery sequence, UUUAAAC, that appears to be even more efficient in promoting −1 PRF than the HIV-1 UUUUUUA motif (41.7% versus 24.7% when placed upstream of a minimal IBV pseudoknot) [19, 25]. Taken together, albeit a pseudoknot structure provides a stronger mechanical hindrance for ribosome progression, an efficient slippery sequence probably works synergistically with a less topologically restrained stem-loop. However, the quantitative relationships between these components, as to why certain sequences are many folds more slippery than others [25], require further investigations.

The above examples demonstrate that single-molecule spectroscopy can straightforwardly access physical quantities that seem to better correlate with pseudoknot-stimulated −1 PRF efficiencies, thereby providing unprecedented mechanochemical details for the underlying mechanisms [16, 31, 32].

## 3. Coarse-Grained Elastic Network Model Provides Further Insights into Global Motions of the Ribosome and May Guide the Labeling Scheme for Single-Molecule Spectroscopy

**Figure 3:**
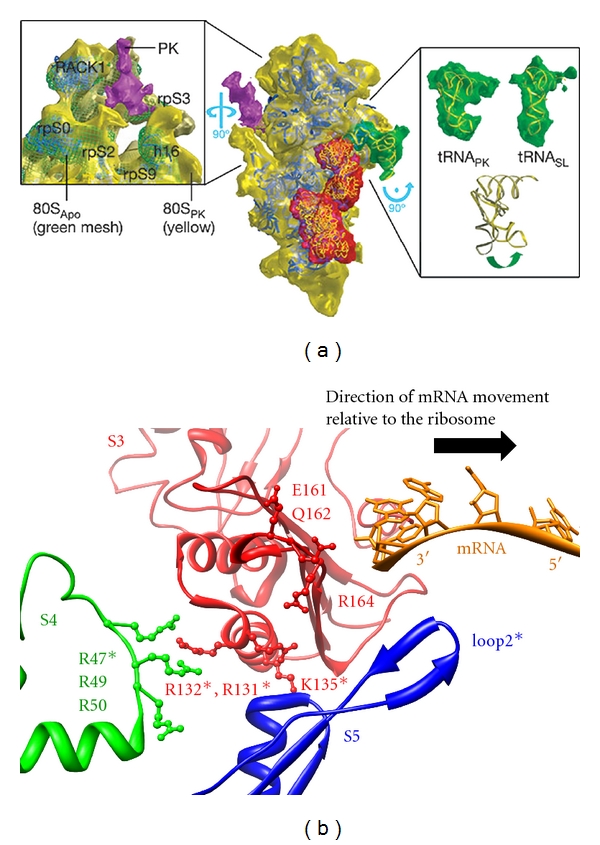
(a) Cryo-EM reconstituted map of pseudoknot-stalled mammalian 40S small ribosomal subunit. The close-up view on the left shows the pseudoknot (PK, purple) binding to the putative ribosomal helicase site, namely, rpS3 (equivalent to prokaryotic S3), rpS9 (S4 in prokaryotes), and rpS2 (S5 in prokaryotes). Compared with the vacant 80S ribosome map (80S_Apo_, green mesh), these subunits lining the mRNA entrance tunnel (yellow) move slightly toward the pseudoknot. The close-up view on the right shows pseudoknot-stalled P-site tRNA (tRNA_PK_) distortion relative to the stem-loop-stalled tRNA (tRNA_SL_). The data implies that although both pseudoknot and stem-loop promote ribosome stalling, only pseudoknot can induce conformational changes in the ribosome complex. Figure reproduced with permission from [44]. (b) Close-up view of prokaryotic mRNA entrance tunnel [45] (PDB 3KNJ). Viewed from interior of the ribosome, residues implicated in interacting with the mRNA by ENM are labeled [46], and shown in ball-and-stick representations. Asterisks denote functional residues reported to be involved in ribosomal helicase activity (residues in S3 and S4) and translational fidelity (loop 2 in S5) by previous experiments [29, 47]. The ribosome reads mRNA in a 5′ to 3′ fashion, that is, opposite to mRNA movement indicated by the black arrow. Messenger RNA, S3, S4, and S5 are shown in orange, red, green, and blue, respectively.

 The ribosome has been shown to possess intrinsic mRNA helicase activity for resolving duplex structures of the mRNA during translation* in vitro* [29]. Interestingly, in addition to the superior stability of pseudoknots arising from tertiary interactions and torsional restraints, it has been proposed that stereochemical mismatch between the pseudoknot structure and the geometry of the putative ribosomal helicase sites would block the entry of downstream mRNA [28, 44]. This view is supported by the fact that some mutations in Loop 2 that are not anticipated to affect tertiary interactions, but are suspected to alter contacts with the ribosome, significantly lower −1 PRF efficiencies [60]. This gives rise to the interesting possibility that other than acting as a general mechanical hindrance, specific interactions between the pseudoknot and the mRNA entry site of the ribosome could promote −1 PRF by inducing conformational changes of the ribosome complex allosterically. Indeed, although both pseudoknots and stem-loops can stall ribosomes, only pseudoknots are able to induce distortion in the P-site tRNA, as observed in the cryoelectron microscopy (cryo-EM) maps of stalled mammalian 80S ribosomes (Figure 3(a)) [44]. The low resolution (16.2 Å), however, was not sufficient for visualizing atomic details of the structural dynamics during frameshifting. Unfortunately, high-resolution single-molecule techniques are also constrained by their pulling or labeling sites and are consequently blind to such global structural changes. Computational modeling can, on the other hand, monitor overall motions of the molecule from detailed structural models.

A coarse-grained elastic network model [61] only takes the C_*α*_ atom for each amino acid residue into account as “nodes” in a protein network [62]. For each nucleotide, 2 or 3 representative atoms are assigned as nodes [63, 64]. The interaction between each node is then approximated by simple harmonic potential, which dictates the fluctuations of these nodes. The nodal vibrations are further decomposed into a number of normal modes, whose contributions to the overall dynamics of a protein are inversely weighted by their respective frequency-squared. As a consequence, the biologically relevant motions of a protein, which generally involve large-scale conformational changes, are dominated by low-frequency modes in ENM [65–67].

ENM is based on the view that protein dynamics is largely determined by the topology of native contacts, as has been proposed and supported by several studies [68–72]. Accordingly, the physical and chemical properties of each residue are not taken into consideration in ENM, and the computational complexity is greatly reduced compared with molecular dynamics simulations [71, 73]. It takes even less time to calculate only the low-frequency modes that dominate macromolecular motions, making ENM especially suitable for modeling large complexes [70]. Indeed, the ratchet-like motion of the ribosome, one of the most important global motions characterizing ribosomal translocation [74], was clearly described by ENM calculations that only took a few slow modes into account [64, 71]. Furthermore, global deformations of the ribosome can be calculated by ENM iteratively based on X-ray structures and then fitted into cryo-EM structures originated from different states of the ribosome bound with various factors, unraveling large-scale conformational changes from inherently low-resolution cryo-EM images [75].

The relationship between accommodation of A-site aminoacyl-tRNA and dissociation of E-site deacylated tRNA remained elusive for years [16, 76]. Based on the ENM simulation results, Wang et al. predicted that the movements of A-site and E-site tRNAs are uncoupled (the orientation correlation is 0.165, where a unity value would indicate perfectly concerted motions) [64]. This prediction has been supported by correlation analysis of a later single-molecule fluorescence experiment (correlation coefficient *r* = 0.04) [76]. Likewise, Kurkcuoglu et al. utilized the same approach to detect possible active sites responsible for the intrinsic helicase activity of the ribosome (Figure 3(b)) [46]. The predictions not only agreed with previous results [29], but also gave more possible key residues that have not been confirmed experimentally. Therefore, ENM, which samples equilibrium dynamics and predicts global conformational changes, can guide the labeling scheme for future single-molecule experiments crucial for probing direct interactions between the ribosomal helicase and the pseudoknot.

 Modeling perturbed ribosomal dynamics induced by pseudoknots can be readily carried out with ENM and linear response theories [77], where the magnitude of perturbation force exerted on the ribosome can be inferred from unwinding forces of pseudoknots provided by single-molecule measurements. Alternatively, the pulling force directly applied on the ribosome by optical tweezers provides another plausible experimental approach for model validations and refinement. This scheme may as well be applied to other supramolecular assemblages.

## 4. Future Perspectives

 Due to the asynchronous nature of stochastic reactions, detailed molecular mechanisms are often difficult to be inferred from conventional bulk experiments. Nevertheless, developments of recent biophysical tools, particularly single-molecule techniques, have elucidated much about the mechanical and functional properties of pseudoknots [31, 32, 41, 44], the action of the ribosomal helicase [46, 78], and the mechanisms of translational machinery as a whole [52, 76, 79, 80]. Indeed, combining data from cryo-EM, X-ray crystallography, as well as molecular dynamics simulations/modeling has provided predictions and insights into the interactions between the ribosomal L1 stalk and tRNAs, which agree well with smFRET results [80]. It is tempting to ask what we can learn by applying similarly combined methodologies to investigations of −1 PRF mechanisms.

Structural, computational, and single-molecule approaches are complementary to each other. Computational modeling and simulations unravel the dynamic nature of molecules and provide physics-based methods for protein deformations in the presence of externally applied forces [69, 73, 81, 82]. The calculations could make “rational” biotin/digoxigenin labeling possible in a single-molecule experiment, by labeling the ribosome at sites that cause the least conformational changes and the resulting dissociation from the mRNA during optical tweezing. The mechanical descriptions thereof can be rather physiologically relevant, as in the case of titin kinase, which serves as a molecular force sensor in muscle cells [81–83]. On the other hand, single-molecule force and fluorescence spectroscopies probe real-time structural transitions, such as folding-unfolding as well as stretching, for proteins [84–86], RNAs [87], and complexes [41, 88]. These data can be subsequently used to validate and/or refine the physics models and molecular simulations.

 Understandings to the translational machinery as well as −1 PRF mechanisms have suggested attractive targets for antiviral therapies. Although it is possible to develop drugs that target the eukaryotic 80S ribosome and alter −1 PRF [89, 90], the side-effects are unclear, owing to potential cellular genes that utilize frameshifting, but have not yet been found [91–93]. Developing drugs that specifically interact with viral −1 PRF-stimulating structures could be a good intervention strategy. Indeed, small ligands have been identified to alter viral −1 PRF efficiencies by binding to the −1 PRF-promoting stem-loop in HIV-1 and the pseudoknot in severe acute respiratory syndrome coronavirus (SARS-CoV) [6, 21, 27]. As described above, single-molecule force spectroscopy can provide the unfolding forces of various RNA structures, which correlate with −1 PRF efficiencies much better than thermodynamic stabilities. Studies with various mutant −1 PRF-promoting structures may facilitate drug discovery by identifying the essential residues and bondings responsible for their mechanical stabilities as well as interactions with the ribosome. Accordingly, combining the new biophysical tools sheds light on how future antiviral agents can be developed to work against the ubiquitous −1 PRF mechanisms among viruses.
